# A cross-sectional survey study of the impact of COVID-19 pandemic on the training and quality of life of Italian medical residents in the Lombardy region

**DOI:** 10.1080/07853890.2022.2105392

**Published:** 2022-08-24

**Authors:** Elena Abati, Leonardo Nelva Stellio, Arianna Manini, Francesco Moroni, Lorenzo Azzalini, Luz Maria Vilca

**Affiliations:** a Department of Pathophysiology and Transplant (DEPT), Dino Ferrari Centre, Neuroscience Section, University of Milan, Milan, Italy; b Department of Women, Mothers and Neonates, Buzzi Children’s Hospital, ASST Fatebenefratelli Sacco, University of Milan, Milan, Italy; c Università Vita-Salute San Raffaele, Milan, Italy; d Division of Cardiology, Department of Medicine, University of Washington, Seattle, WA, USA; e Unit of Obstetrics and Gynecology, Buzzi Children’s Hospital, ASST Fatebenefratelli Sacco, University of Milan, Milan, Italy

**Keywords:** SARS-CoV-2, internship and residency, clinical competence, education, research activities, cross-sectional studies, personal satisfaction

## Abstract

**Introduction:**

The reorganization of the healthcare system prompted by severe acute respiratory syndrome coronavirus 2 (SARS-CoV-2) pandemic has posed unique challenges for Residency Training Programs worldwide. To mitigate its potential negative effects, it is crucial to assess how the pandemic influenced the activity and quality of life of residents. The purpose of this study was to assess the impact of the pandemic on residents’ competencies, satisfaction, working load, training patterns and occupational exposure in the clinical, surgical, research and didactic fields and to quantify its effects on quality of life and risk perception.

**Methods:**

An online cross-sectional survey was distributed between 1 June 2020 and 31 July 2020 to 1645 residents enrolled in all Residency Programs of four Universities in northern Italy. The survey included questions about clinical, surgical, and research competencies, educational activity, and quality of life pre- and post-pandemic, and on policies and workplace interventions to reduce exposure to SARS-CoV-2. The main outcome measure was the variation in self-perceived clinical, surgical and research competencies and in specialistic training. Data were analysed using the statistical package R Core Team 4.0.0, estimating mean and standard deviation or median and interquartile range for continuous variables. Variables were compared using chi-square test, Fisher exact tests or McNemar test, as appropriate.

A multivariate binary logistic regression analysis was performed to test the effect of different factors on the impact of coronavirus disease-2019 (COVID-19) on self-perceived clinical and research competencies and on didactic training.

**Results:**

A total of 498 residents completed the survey (response rate 30.3%). The mean age of respondents was 28.9 years, 62.9% were women, and 52.4% were enrolled in the first two years of Training Programs. On the first pandemic wave, over 60% of residents reported a negative impact of the pandemic on their specialistic training. In contrast, 40% of residents involved in clinical duties perceived an improvement in their clinical competences, especially those involved in COVID-19 care, and 34.5% perceived an improvement in their research competences, particularly junior residents, while only 3.5% reported an improvement in surgical skills. Most surgical residents (88.5%) reported a decrease in surgical activities, mainly due to reduced hospital bed capacity and reduction of elective surgery. Almost 90% of all residents experienced a reduction in their didactic activities, but 80% stated their Residency Program adopted virtual training methods. A statistically significant reduction in all examined quality of life items post-pandemic vs. pre-pandemic was found. Even though most survey participants reported the availability of personal protective equipment for residents, 44% considered themselves to be at higher risk of exposure compared to senior staff.

**Conclusion:**

COVID-19 pandemic caused a significant disruption in surgical training, but it had a positive impact on clinical competencies among residents involved in COVID-19 and urgent care. The pandemic had a detrimental effect on all quality of life aspects, and most residents considered themselves at higher risk of SARS-CoV-2 infection compared to other healthcare professionals.

**Key Messages**
- Coronavirus disease-2019 (COVID-19) pandemic caused a significant disruption in surgical training, but it had a positive impact on clinical competencies among residents involved in COVID-19 and urgent care.
- Most residents experienced a reduction of didactic activities. Although the majority of training programs implemented virtual training methods to counteract the restrictions imposed by the pandemic, only half of the residents were satisfied of them.
- A vast proportion of residents had a high occupational exposure to SARS-CoV-2 and considered themselves at higher risk of COVID-19 infection compared to senior staff.
- The survey highlighted a statistically significant reduction in five key quality of life measures (i.e. sleep, mood, familiar relationships and social relationships quality and employment satisfaction) during the first wave, with mood and social relationships being the most affected. Notably, employment satisfaction was significantly higher in medical compared to surgical residents.

## Introduction

The World Health Organization (WHO) officially declared the severe acute respiratory syndrome coronavirus 2 (SARS-CoV-2) pandemic on 11 March 2020. Italy was the first European country to detect and suffer from massive clusters of coronavirus disease-2019 (COVID-19) infections, which resulted in a tremendous disruption of the national health system [1]. During the first wave, local hospitals were forced to make drastic changes to reorganize spaces and workforce and provide care for the huge number of severely ill patients needing respiratory support. Unsurprisingly, many physicians witnessed major changes in their activity, including medical and surgical residents.

SARS-CoV-2 has had a large impact on the medical education of residents. During the pandemic, different studies reported several changes in postgraduate medical training worldwide, such as reduction of workload and of operative and procedural volume [2–9], interruption of scheduled rotations [2,4,10], adoption of telemedicine services and remote learning [2,4,9,11–14], increased work hours [15], growing concerns over professional development and career goals [9,15,16], and an overall negative impact on clinical education [4,6,10,17].

In addition to its effects on physical health, the pandemic deeply impacted social, psychological, and economic well-being as well [18]. Disruption to work and social life possibly affected also the quality of life and job satisfaction of trainees, as it has already been pointed out in several studies concerning the mental health of health care workers (HCWs) during the pandemic [19–22]. Several surveys of medical trainees showed worsened quality of life and work-life balance [14,17,23] and an increase in reported stress, anxiety, burnout, and/or depression [5,6,8,14–16,24]. However, the effects of the pandemic may differ according to the geographical area, due to differences in contagion rate, governmental response, preparedness of health services, and cultural differences. As regards Italian residents, some surveys already analysed the impact of SARS-CoV-2 pandemic on trainees of specific programs [4,10,17,25–31], showing an overall disruption of training organization. Data regarding quality of life were provided only by two studies, which reported an overall negative impact [17,32]. Many programs tried to maintain educational activities by transitioning lessons into a virtual modality and implementing telemedicine services [4,6,9,33]. However, the profound differences between residency programs should be taken into consideration while evaluating these results and the efficacy of the adopted measures. Given the lack of uniformity of the above studies, it is difficult to compare them, and transversal studies exploring the effects of the pandemic on residents from different specialties are needed for this purpose. A few international studies of this kind have already been published [6,15,34], showing higher rates of anxiety and burnout in trainees with greater exposure to patients with COVID-19 [15], female trainees [6,24] and trainees aged <39 years [24]. A disruption in training organization [6] and an overall decrease in the quality of education [34] was reported, especially by senior trainees [6]. A systematic review and meta-analysis of published studies on this topic, released in December 2021, pointed out that surgical specialties were probably the most affected, even though there is a disproportion in the available data and effects on medical specialties were rarely reported [35]. Another potential source of bias was that over half of available studies have been conducted in the United States [35]. In addition, none of the screened studies has compared the learning outcomes of residents between pre-pandemic and pandemic periods [35].

Despite the insights given by previous works, many authors feel that further research is needed in order to evaluate the effects of the pandemic on the career of residents from different fields and different countries, and to implement methods for maintaining the quality of medical education during future crises [34,35].

With the present study, we aimed to fill this gap. This is the first Italian study to depict the effect of the virus on a wide group of residents of different medical and surgical specialties. The purpose of this study was to investigate the changes in competencies, workload, training patterns, occupational exposure, and quality of life experienced by medical and surgical residents during the first wave of COVID-19 pandemic in Lombardy, the COVID-19 worst-hit Italian region.

## Methods

### Study design

This cross-sectional survey study was designed to obtain information from medical residents enrolled in the Residency Programs of four Italian Universities of the Lombardy area. We followed the Strengthening the Reporting of Observational Studies in Epidemiology (STROBE) reporting guidelines and the Consensus-Based Checklist for Reporting of Survey Studies (CROSS) guidelines. The study was reviewed and approved by the local institutional review board (name of the ethical committee “Comitato Etico di Milano Area 2”, n. 663_2020bis). The survey was conducted from 1 June 2020 to 31 July 2020. Survey participation was voluntary and anonymous, and the participants were asked to give informed consent at the beginning. No economical compensation was offered to participants.

### Conceptual framework

This study used a questionnaire-based approach to obtain information from medical residents enrolled in the Residency Programs of four Italian Universities of the Lombardy area. Lombardy was the first Italian region to detect COVID-19 cases and during the pandemic first wave had to face a massive amount of cases with severe disruption of health services.

Postgraduate medical training in Italy consists of a 4- or 5-year Residency (according to the chosen specialty) following on from a national exam. Admission to the Residency Program is contingent on test results and overall academic scores, and there is a capped annual number of residency positions. During training, residents usually work in academic hospitals, gaining clinical experience in their relevant specialty and rotating throughout the main subspecialties. In addition, they follow an educational program mainly based on frontal lessons (i.e. teaching activities which take place from the front of the classroom) and they may be involved in research projects. The level of autonomy with which trainees can perform the required tasks is regulated by law and depends on the PGY. In general, trainees of the first two years cannot carry out tasks in complete autonomy, while trainees of the last years are more independent. As said, Training Programs in Italy are regulated by national guidelines that report the educational goals for each training year, but the methods for assessing activities and skills are very heterogeneous among the different Universities and mainly rely on paper logs. For this reason, we chose to use a survey based on subjective questions. We chose to host it on an online platform to ensure speed and ease of use.

21 February 2020 was chosen as the reference date because the first COVID-19-related death in Italy has been registered on that day. This date is regarded as the beginning of the so-called “first wave” of COVID-19 pandemic in Italy; infection rate then started to subside at the end of May and during June 2020 [36]. The period from 1 June 2020 to 31 July 2020 can be regarded as the end of the COVID-19 first wave in Italy.

### Measurement tools

An online-based questionnaire was designed using the collaborative web survey software Google Forms to investigate the impact of COVID-19 pandemic on Italian medical residents’ competencies, working load, training, occupational exposure, and quality of life. The authors EA and LNS (last-year residents experienced in research studies) and LMV (senior statistician with experience in the conduction of survey studies) developed the survey after literature review about medical education, residency training, occupational exposure, and mental health among HCWs, and with feedback from Chiefs and Program Directors of their hospitals to assess the content and face validity of the questionnaire. A combination of binary (yes-no), multiple choice, 5-point Likert-type scale and ranking questions were employed. The complete questionnaire can be found in Supplementary Material (Appendix 1). Briefly, the survey was composed by 95 questions, divided into seven sections:

1. Demographic features, including information about participants’ age, social and economic status, lifestyle, type and location of the Residency Program (region, city, University, hospital).
2. Clinical training, assessing clinical duties of trainees before and after 21 February 2020.
3. Surgical training, assessing surgical duties of trainees before and after 21 February 2020.
4. Research training, assessing research work of trainees before and after 21 February 2020.
5. Training abroad, assessing whether the trainee was working/studying abroad before 21 February 2020 and pandemic’s effects on hosting structure activities and permanence abroad.
6. Didactics, investigating the effects of COVID-19 pandemic on education curricula, adoption of telemedicine strategies and residents’ satisfaction regarding education curricula before and after 21 February 2020.
7. Contagion prevention and surveillance, exploring contacts with confirmed SARS-CoV-2 cases, preventive measures taken by Institutions, adequate supply of personal protective equipment (PPE), and special training received by residents working in COVID-19 wards.
8. Quality of life, investigating trainees’ lifestyle, mood, personal and social relationships, and job satisfaction before and after 21 February 2020.

The main outcome measures were variation in self-perceived clinical, surgical and research competencies and in specialistic training. These outcomes were assessed with a 5-point Likert-type scale assessing the level of self-perceived competence before and after the onset of the pandemic (21 February 2020). Secondary outcome measures included changes in working load and in satisfaction regarding clinical, surgical, research and didactic activities and variation in quality of life items before and after the onset of the pandemic; reported adoption of virtual education strategies; perception of increased risk of COVID-19 infection and availability of personal protective equipment (PPE) among residents compared to other HCWs.

The purpose and objectives of the study and the composition of the study group were described to all participants in the online survey homepage. The survey was pilot-tested with a convenience sample of 10 residents to ensure clarity and ease of administration.

### Data collection procedure

We invited to participate in the study all the residents enrolled in the Residency Programs of four Italian Universities (1000 residents from “Università degli Studi di Milano”, 389 from “Università Vita-Salute San Raffaele”, 206 residents from “Università Milano-Bicocca”, 50 residents from “Università degli Studi di Pavia”).

An invitation to participate in this cross-sectional study, accompanied by the link to the online survey, was sent *via* e-mail to the residents by the respective Training Program offices and by the residents’ representatives. Three reminder emails were sent to participants until study closure to optimize response rates. Limit of one response per participant was ensured. After the closing date for questionnaires’ submissions, the results were downloaded as a CSV (comma-separated values).

### Data analysis

Data were analysed using the statistical package R Core Team, version 4.0.0 (R Foundation for Statistical Computing, Vienna, Austria, 2013).

Response frequency distributions were tabulated for each question, excluding non-responses from the denominators. Mean and standard deviation or median and interquartile range were estimated for continuous variables. Cross-tabulated frequencies and percentages were computed, and chi-square test or Fisher exact tests were performed to compare categorical variables, as appropriate. McNemar test was used to compare the answers to pre-pandemic vs. post-pandemic questions about quality of life and training satisfaction asked to the medical residents.

The effect of different factors on the impact of COVID-19 on self-perceived clinical and research competencies and on didactic training was tested using a multivariate binary logistic regression analysis. Surgical competence was excluded from this analysis because of the presence of null values in some columns. Collinearity and interactions between variables were checked. All statistical analyses and interval estimates for odds ratio (OR) were two-tailed and performed using an alpha error = 0.05. A value of *p* < .05 was considered significant.

During data analysis, we decided to separate the University of Milan from the other Universities of Lombardy because Milan was less affected by COVID-19 during the first spreading of the pandemic compared to other cities of Lombardy (i.e. Pavia, Bergamo). Accordingly, the hospitals of the city of Milan (i.e. where Residents can work during their Training Program) faced COVID-19 breakdown later and to a lesser extent compared to those of the other cities of Lombardy.

## Results

### Residents’ and training programs’ characteristics

Out of 1645 Italian Residents who received the online survey, a total of 498 completed the questionnaire (response rate 30.3%). The mean age of respondents was 28.9 years (SD 2.1), and 313 (62.9%) were women (Table 1). Most respondents (332, 66.7%) were less than 30 years old (approximately representing the age of Residents at half of their Training Programs). Junior (Program year – PY − 1 and 2) and senior (PY 3-4 and, when applicable, 5) residents were almost equally represented (52.4% vs. 47.6%), while medical residents were starkly overrepresented compared to the surgical ones (73.3% vs. 26.7%) (Table 1). At the time of the survey, most participants (93.4%) were working in Lombardy, the worst-hit Italian region during COVID-19's first wave (Table 1). Most subjects were declared to be single (440 out of 498), and only one was divorced (Table S1 in Supplementary Material). Therefore, we decided to combine these two groups in the following analysis due to the similar legal profile in Italy and, often, living conditions. Complete respondents’ demographics and detailed information regarding involvement in clinical, surgical, research and abroad activity before and after the inception of the pandemic is provided in Table S1 in Supplementary Material.

**Table 1.** Sociodemographic and training program characteristics of surveyed Italian residents.

Characteristic	No. (%) (*n* = 498)
Age category (in years)	
<30	332 (66.7%)
> =30	166 (33.3%)
Sex	
Female	313 (62.9%)
Male	185 (37.1%)
University	
University of Milan	377 (75.7%)
Other universities	121 (24.3%)
Workplace region	
Lombardy (Milan)	381 (76.5%)
Lombardy (other cities)	84 (16.9%)
Other regions	33 (6.6%)
Residency Program	
Medical and other Specialties	365 (73.3%)
Surgical specialties	133 (26.7%)
Training program year	
Junior (PY1-2)	261 (52.4%)
Senior (PY3-4-5)	237 (47.6%)

### Main outcome measures

Regarding self-perceived clinical competencies, 174 (40.5%) residents out of 430 involved in clinical activity believed that their competencies had not changed during COVID-19 first wave, 172 (40%) considered they had improved, and 84 (19.5%) that they had worsened. A significantly higher proportion of medical residents perceived an improvement in their clinical competencies compared to surgical residents (47.1% vs. 20.7%, *p* < .0001). No significant differences between junior and senior residents were detected.

Notably, residents of clinical, surgical or other non-COVID-19-related residency programs were less likely to perceive an improvement in their clinical competencies [odds ratio [OR] = .41 (95% confidence interval [CI] 0.24–0.70); OR = 0.15 (95% CI 0.08–0.28); OR = 0.15 (95% CI 0.05–0.39), respectively] compared to residents of COVID-19-related residency programs (Pneumology, Infectious Diseases, Critical Care or Emergency Medicine) (Table 2).

**Table 2.** Factors associated with improvement in clinical and research competencies and in didactic training among surveyed residents in Italy during COVID-19 pandemic first wave (univariate and multivariate analysis).

	Clinical competencies	Research competencies	Didactic training
Predictor	aOR (95% CI)	aOR (95% CI)	aOR (95% CI)
Age group (years) [ref: 30–40]			
30–40	0.90 (0.52–1.55)	1.34 (0.70–2.49)	1.56 (0.72–3.40)
Sex [ref: female]			
Male	1.07 (0.68–1.69)	1.20 (0.70–2.05)	0.72 (0.36–1.38)
Civil status [ref: single or divorced]			
Married	1.92 (0.96–3.87)	1.15 (0.48–2.64)	0.44 (0.12–1.28)
Annual family income [ref: <2500 €]			
2500–3500 €	0.66 (0.37–1.16)	0.64 (0.32–1.25)	1.29 (0.56–2.76)
>3500 €	0.80 (0.42–1.48)	1.11 (0.54–2.26)	1.50 (0.63–3.33)
University [ref: other universities]			
University of Milan	0.59 (0.33–1.07)	1.47 (0.71–3.10)	0.24 (0.11–0.54***)
Workplace region [ref: Lombardy (Milan)]			
Lombardy (other cities)	2.24 (1.20–4.6**)	2.03 (0.89–4.64)	0.57 (0.22–1.43)
Other regions	1.28 (0.50–3.24)	1.31 (0.32–4.57)	1.96 (0.67–5.20)
Type of residency program [ref: COVID-related specialties^§^]			
Clinical specialties	0.42 (0.24–0.70**)	1.59 (0.78–3.29)	1.57 (0.69–3.79)
Surgical specialties	0.15 (0.08–0.28***)	0.76 (0.34–1.00)	1.38 (0.55–3.00)
Other specialties^∞^	0.15 (0.05–0.39***)	1.09 (0.43–2.72)	1.48 (0.45-4.50)
Training program year [ref: junior (PY1-2)]			
Senior (PY3-4-5)	0.82 (0.50–1.34)	0.45 (0.24–0.81**)	0.97 (0.47–1.99)

aOR: adjusted odds ratio; CI: confidence interval; *N*: number; Ref.: reference category.

**p < 0.01 and ***p < 0.001.

Concerning self-perceived surgical competencies, 74 (60.7%) out of 122 residents involved in surgical activity reported that their competencies had worsened during COVID-19 pandemic, 44 (36%) that they were unchanged and only 4 (3.5%) that they had improved. No statistically significant differences were found between junior and senior residents.

As for self-perceived research competencies, 113 (39%) out of 288 residents involved in research activity declared their research competencies had not changed during COVID-19 first wave; 100 (34.5%), that they had improved; and 75 (26%), that they had worsened. The proportion of junior residents who perceived an improvement in their research competencies was significantly higher than senior residents (42.6% vs. 27.2%, *p* = .009), while we did not observe significant differences according to the type of residency. Accordingly, in the multivariate model, senior residents were less likely to perceive an improvement in their research competencies compared to junior residents [OR = 0.45 (95% CI 0.24–0.81] (Table 2).

When asked about the perception of COVID-19 outbreak impact on their education, 325 (65.3%) out of 498 responders declared that it had worsened, 124 (24.9%) that it had not changed and 49 (9.8%) that it had improved. Responses did not differ significantly between medical and surgical residents, nor between junior and senior residents. Interestingly, residents belonging to the University of Milan were significantly less likely to report an improvement compared to other universities [OR = 0.24 (95% CI 0.11–0.54)] (Table 2).

### Secondary outcome measures

#### Impact of COVID-19 on clinical, surgical, research, and didactic activity

Among the 272 trainees involved in inpatient care, 193 (48.5%) experienced an increase in their clinical inpatient activity during COVID-19 first wave, while 166 (41.7%) experienced a reduction and 39 (10.0%) did not report any change. Residents involved in outpatient clinics or day hospital services reported a more dramatic COVID-19 impact, as a reduction in these activities was declared by 81.1% and 77.1% of trainees, respectively.

Indeed, 272 (63.3%) out of 430 trainees declared that they faced a modification in the type of clinical activity conducted during pandemic; in 55.9% of cases, they were deployed to COVID-19 wards. In half of cases, this deployment was not voluntary. Medical residents were more subjected to change in their activities compared to surgical residents (63.3% vs. 55.0%).

Among the 122 residents involved in surgical activities, 108 (88.5%) observed a reduction in their surgical activities during COVID-19 first wave, while 5 (4.1%) experienced an increase and 9 (7.4%) did not report any change.

Among the 288 residents involved in research activities, 160 (55.6%) residents experienced an increase in research activity, 85 (29.5%) experienced a reduction and 43 (14.9%) did not report any change.

After the onset of the pandemic, 444 (89.2%) residents out of 498 experienced a decrease in educational activities, 38 (7.6%) stated that there was no change and 16 (3%) experienced an increase (Table S3 in Supplementary Material). Three hundred and ninety-one (78.5%) residents said that their residency programs adopted virtual training methods after the start of the pandemic. Characteristics of virtual training were similar among medical and surgical residents (*p* > .05).

The changes in clinical, surgical and research working load and in didactic training patterns among residents, as well as their main causes are shown in Table S3 in Supplementary Material.

#### Satisfaction regarding clinical, surgical, research and didactic activity

**Figure 1.**
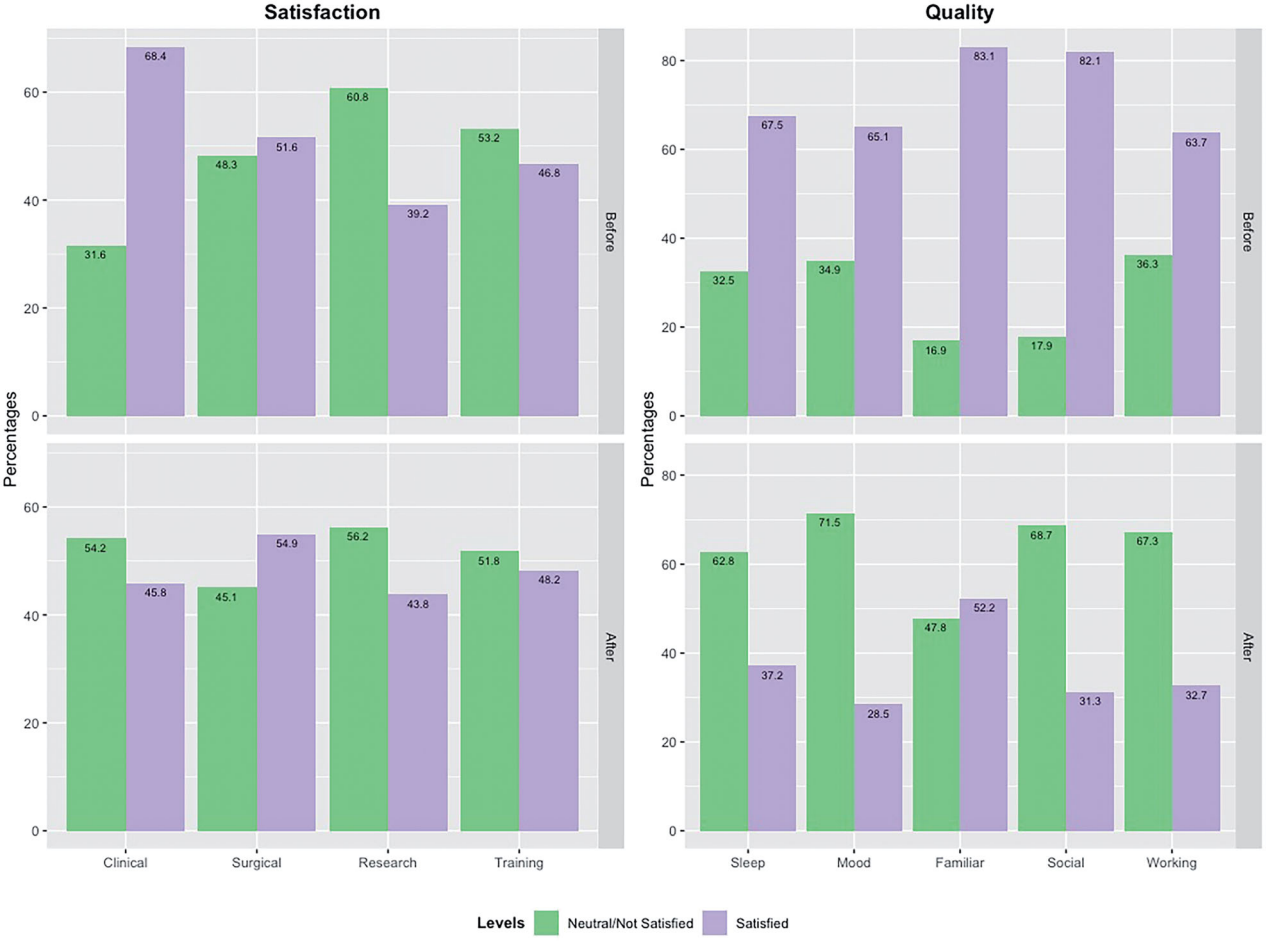
Satisfaction and quality of life of residents before and after COVID-19 first wave. (A) Satisfaction regarding clinical, surgical, research and didactic activities before and after COVID-19 first wave. (B) Satisfaction regarding sleep, mood, familiar and social relationships, and employment before and after COVID-19 first wave (sleep: *p* < 0.0001; mood: *p* < 0.0001; familiar relationships: *p* < 0.0001; social relationships: *p* < 0.0001; employment satisfaction: *p* < 0.0001).

We assessed perceived variation in residents’ satisfaction on their clinical activity before and after the COVID-19 pandemic. We found that, while 68.4% declared that they were satisfied with their clinical activity before COVID-19, this percentage dropped to 45.8% when asked about their satisfaction after the onset of the pandemic (*n =* 430; *p* < .0001; Figure 1). We found a statistically significant difference in satisfaction on clinical activity after the pandemic between medical versus surgical residents (50.3% satisfied vs. 33.6%, *p* = .003). No statistically significant difference was found between junior and senior residents.

Conversely, the satisfaction reported by residents on their surgical activity did not change significantly, moving from 51.6% of residents reporting satisfaction before COVID-19 to 55% reporting satisfaction after the COVID-19 pandemic (*n =* 122; *p* > .05; Figure 1). No statistically significant difference was found between junior and senior residents.

The proportion of residents who stated they were satisfied with their research activity before and after the COVID-19 pandemic rose from 39% to 44% after COVID-19 first wave (*n =* 288; *p* > .05; Figure 1). No statistically significant differences were found according to type of residency nor year of training.

The proportion of residents who stated they were satisfied with their didactic training before and after the COVID-19 pandemic remained constant, moving from 46.8% to 48.2% (*n =* 498; *p* > .05; Figure 1). Among residents who received virtual training during the pandemic (*n* = 391), 197 (50.4%) stated that they were satisfied with their current virtual didactic activities. No statistically significant differences were found according to the type of residency nor to the year of training.

#### Quality of life

Overall, respondents stated that there had been a decrease in their quality of life among all the items assessed (Figure 1). We found a statistically significant difference between individual pre-pandemic and pandemic periods scores for each item. Particularly, mood and social relationships were the most affected.

More medical residents compared to surgical residents were satisfied with their employment (38% medical residents vs. 18% surgical residents, *p* < .0001). No statistically significant differences were found for other items among medical vs. surgical residents nor among junior vs. senior residents.

#### Contagion prevention, safety and surveillance

Three hundred and fifty-five (71.3%) residents reported contact with confirmed SARS-CoV-2 cases (patients or colleagues), with a statistically significant prevalence among surgical versus medical residents (79% vs. 68.5%, *p* = .03). As preventative measures, 132 (37.1%) participants underwent nasopharyngeal swab, 113 (31.8%) were advised to start quarantine only in case of appearance of COVID-19-related symptoms, 40 (11.2%) were asked to start precautionary home isolation regardless of the presence of symptoms and 24 (6.7%) underwent serological analyses for the presence of SARS-CoV-2 IgG/IgM. In 109 cases (30.7%), the hospital/hosting structure did not take any precautionary measures.

Among surveyed residents, 99 (20%) experienced symptoms suggestive for COVID-19; in most cases the symptoms were mild (83 residents, 83.8%), in some cases (16, 16.2%) they were moderate but did not require hospitalization.

As regards the availability of PPE, 413 (82.9%) residents reported that adequate PPE were available for them on all occasions or for >75% of time, 53 (10.6%) said they were available for 25% to 75% of time, 32 (6.4%) for <25% of time or never. Notably, 220 (44.2%) out of 498 considered themselves at higher risk of contracting COVID-19 infection compared to other health care professionals. Moreover, more junior residents agreed with this statement compared to their senior counterparts (49.8% vs. 38%, *p* = .01). When asked whether they had the perception that PPE was not administered equally to residents compared to other physicians, 353 (70.9%) residents said this disparity happened rarely or never, 84 (16.9%) sometimes, 61 (12.2%) very often or always.

Complete data regarding infection prevention and control practices undertaken by local Institutions and Training Programs during the COVID-19 pandemic and infection prevention measures adopted by residents outside the hospital setting are reported in Table S3 in Supplementary Material.

## Discussion

With our survey, we found that COVID-19 first wave has had a significant impact on the acquisition of competencies of Italian residents and on their workload, training patterns, occupational exposure, and quality of life. Noteworthy, the effects of COVID-19 did not follow a homogeneous path of variation, but rather exerted different effects according to the training program and type of activity.

Most surveyed residents (60%) reported a negative impact of the first pandemic wave on their specialistic training. Among residents involved in clinical duties, 40% perceived an improvement in their clinical competencies, especially those involved in COVID-19 care, while only 3.5% of surgical residents reported an improvement in surgical competencies, and most (88.5%) reported a decrease in surgical activities. Most residents (90%) experienced a reduction in their educational activities, but 80% stated that their Residency Program adopted virtual training methods. A statistically significant reduction in all examined quality of life items was found. Even though most survey participants reported availability of PPE for residents, 44% considered themselves to be at higher risk of exposure compared to senior staff.

### COVID-19 pandemic had an overall negative impact on surgical residents and a positive impact on residents enrolled in COVID-related training programs

As mentioned above, less than 5% of the residents involved in surgical activities reported an improvement in self-perceived surgical competencies. The reasons for the negative impact among surgical residents may be explained by the dramatic decrease in surgical activities identified in our study. Indeed, 88.5% of residents involved in surgery reported a reduction in surgical activities, mostly because of ward closures or reduction in bed number. Our results are in line with two previously published systematic reviews [35,37] that found a reduction of operative volume and experience for surgical residents worldwide following COVID-19 outbreak.

Conversely, residents involved in clinical duties (inpatient, outpatient, day hospital and emergency care) reported a less uniform impact following the pandemic onset. A minority of residents involved in clinical duties perceived an improvement in their clinical competence, and, intriguingly, enrolment in a COVID-related Residency Program significantly increased the odds of being part of this group. Indeed, 41.7% of residents involved in inpatient care declared a reduction of clinical activity, largely due to reduced hospitalizations or available beds, while 48.5% reported an increase, mostly because of deployment to a COVID-19-dedicated ward. When considering only the residents involved in outpatient and Day Hospital services, the percentage of participants who experienced a reduction in routinary activity plummeted to 81.0% and 77.0%, respectively. Accordingly, the absolute number of residents involved in inpatient, outpatient, and day hospital care almost halved after the start of the pandemic. It is important to underline that this phenomenon was not observed in Emergency Care, where the absolute number of residents did not vary significantly. In addition, 63% of residents involved in clinical duties reported a modification in the type of their activities. Specifically, 56% of them were deployed to COVID-19 wards and, in half of cases, this deployment was not voluntary. The systematic review performed by Chen and colleagues points out that the effect of COVID-19 on clinical specialties has been rarely mentioned in the literature [35]. Thus, our survey offers an important insight in this regard, showing that the disruption has been less dramatic compared to surgical specialties, and a positive outcome was reported by residents enrolled in specific programs.

Even though the clinical activity among residents did not experience such a dramatic reduction compared to surgical activity, when residents were questioned about their satisfaction on their clinical activities pre and post-pandemic, we identified a satisfaction decrease from 68.4% to 45.8%. Moreover, surgical residents were less satisfied than medical residents with their clinical activities after the onset of the pandemic (50.3% vs. 33.6%). Surprisingly, satisfaction regarding surgical activities did not vary after the onset of the pandemic. Although this result may appear odd, it might be explained by a generally low satisfaction of Italian surgical residents for surgical activities [38], or by a recall bias. Satisfaction regarding research activities soared slightly and a significant percentage of residents involved in research (55.8%) faced an increase of activity, mainly due to the establishment of COVID-19-related research studies. Subjective satisfaction regarding clinical, surgical and research activities has not been assessed in previous studies; our results seem to confirm the findings concerning activities and skill acquisition that we discussed in the previous section.

Taken together, these results suggest that, intuitively, COVID-19 forced a reorganization of training with a dramatic involvement of procedure-based specialties, such as surgical programs, as shown by other studies conducted in the USA [6,39], and non-urgent activities, such as outpatient clinics and day hospital services. At the same time, residents caring for COVID-19 patients witnessed an intensification of their activity, with potential benefits, such as the birth of novel research studies and improvement in clinical competencies, but also increased risk for stress and burn-out, and safety hazards.

### COVID-19 pandemic caused a reduction in educational activities, but most residents reported an implementation of remote learning activities within their training programs

As concerns educational curricula, most residents experienced a reduction of didactic activities. Nevertheless, COVID-19 pandemic offered the opportunity to explore innovative modalities of didactics, commonly known as “distance or remote learning” [40]. Indeed, most surveyed residents reported that their training program implemented virtual training methods to counteract the restrictions imposed by the pandemic. Virtual learning includes online lessons, webinars, and creation of remote or web-based learning modules and was shown to be a viable option for training programs [6]. Virtual education was already known and used worldwide also before the pandemic, but the curricula of many Training Programs in Italy was still based on in-person lessons. It is a current matter of debate whether these strategies should persist also when COVID-19 emergency will subside [41,42]. In the context of graduate medical education, virtual learning, especially asynchronous strategies, may represent a useful method to enhance trainees’ attendance without colliding with busy schedules and duty-hour restrictions [43]. On the other hand, the absence of face-to-face contact may also cause disengagement and reduce teaching effectiveness. Outdated technology and lack of bandwidth, sufficient financial resources, and skills were identified as potential obstacles to its adoption [43–45]. Previous studies attempted to identify solutions, such as user-friendly tools, increased funding for technological equipment and development of e-learning, dedicated training, and the use of interactive methods [43,45]. In our survey, only half of the residents were satisfied with virtual training provided by their Residency Program. In the future, it could be relevant to better investigate e-learning effectiveness and limitations in residency programs.

### Decreased quality of life and job satisfaction, and high COVID-19 exposure among trainees in the Lombardy region

With regard to quality of life, the results of the survey showed that there was a statistically significant reduction in sleep, mood, familiar relationships and social relationships quality and employment satisfaction during the first COVID-19 wave. Mood and social relationships were the most affected. We also found that there was a statistically significant difference in employment satisfaction between medical and surgical residents, a finding that is in line with the consistent reduction in surgical competencies and activity. Surely, isolation measures (i.e. lockdown) as well as increased workload contributed to the observed reduction in quality of life. Different studies conducted among HCWs during the SARS-CoV-2 outbreak found that a significant proportion of participants experienced mild to severe stress, anxiety, depression, and insomnia symptoms [21,22,46]. Female workers, front-line HCWs, younger medical staff, and workers in areas with higher infection rates reported more severe degrees of all psychological symptoms than other HCWs [20,22,46]. Few studies previously investigated the effect of pandemic on residents’ quality of life, showcasing the presence of worsening anxiety and depression [15,24], reduced sleep quality and difficulty concentrating [6,25,26,47], increased risk for burnout, worsened quality of life and work-life balance [14,17,23]. Only a few Italian studies assessed the impact of COVID-19 on the quality of life of Italian residents [17,32]. A study on cardiology fellows reported a negative impact on the psycho-physical wellbeing in 86% of responders. Another study conducted on trainees working in Intensive Care Units and Emergency Departments concluded that 34.3% of participants presented a probable diagnosis of post-traumatic stress disorder (PTSD), while 21.5% presented subclinical PTSD [32]. Our results, showing a significant negative impact on quality of life among residents, should raise concern as they may suggest that Italian residents could be at risk of developing anxiety or mood disturbances in the long term.

Long-term PTSD has been previously observed in emergency services personnel after exposure to critical events [48] and was detected in 14% of HCWs involved in COVID-19 pandemic in a U.S. study [22]. Medical residents were found to present a 3-fold increased risk of developing PTSD symptoms compared to the general population [49]. Based on previous experiences and on the results of our survey, it can be hypothesized that the pandemic crisis might have led to precocious and protracted burnout symptoms, with a subsequent risk of increased drop-out from medical education and medical career. However, our study was time-limited, so that long-term psychological consequences of COVID-19 pandemic could not be assessed.

In previous studies, anxiety and burnout risk was greater in trainees with greater exposure to patients with COVID-19 [15,32]. Indeed, we highlighted that a consistent proportion of residents has been exposed to confirmed positive SARS-CoV-2 cases at work, with divergent actions undertaken by local institutions, and 1 out of 5 experienced COVID-19 symptoms. Even though most survey participants reported availability of PPE for residents, a significant proportion considered themselves at increased risk of contracting COVID-19 infection compared to other health care professionals during COVID-19 first wave, probably due to continuous contacts with confirmed SARS-CoV-2 cases and scarce adoption of preventive measures, such as nasopharyngeal swab. Therefore, our results indicated that residents could have been forced to adopt precautionary measures to avoid transmission in their own household, which might have contributed to the negative impact of COVID-19 first wave on their quality of life.

Literature regarding strategies to tackle trainees’ stress and burnout during and after health crises is still limited. In a Chinese study, resilience and active coping were positively correlated with the quality of life among front-line HCWs [50]. Therefore, interventions aimed at increasing resilience and coping among medical residents could be effective. In spite of that, a recent systematic review pointed out that there is a lack of evidence regarding optimal interventions to support the resilience and mental health of frontline HCWs during or after disease outbreaks, either epidemics or pandemics [51].

### Trainees reported heterogenous surveillance measures and PPE availability

Surveillance among medical trainees is a pivotal public health issue because young HCWs represent an important source of transmission. However, despite >70% of participants stating that they were exposed to confirmed COVID-19 cases, the responses of hosting structures were very heterogeneous, and no preventative measures were undertaken in 30.7% of cases. The availability of PPE remained erratic in some cases. The reason may lie in the nationwide PPE shortage at the beginning of the pandemic, or in the insufficient capacity to process specimens. A potential solution could be to minimize exposure, by reducing the medical personnel who directly interact with patients to the minimum necessary. In case this would not be feasible, guidelines on the use of PPE should be strictly followed, and no distinction should be made between trainees and other HCWs.

### Limitations

Our study is also subject to limitations. Although our aim was to highlight COVID-19 effects on a national scale, Residency Programs present profound regional variability. Furthermore, the burden of COVID-19 was different among Italian regions, with the northern ones being most affected during COVID-19 first wave. As most of our data included Universities located in Lombardy (northern Italy), our results may differ from other Italian or European regions and they cannot be generalized to all Residency Programs in Italy. However, as Lombardy was one of the worst-hit regions by COVID-19 pandemic, our findings provide substantial evidence of how residents experienced a high workload and training disruption. Moreover, as said, our survey was led during COVID-19 first wave. As a consequence, successive data were not available to follow up the effects of COVID-19 pandemic on Italian Residency Programs in the last months and to evaluate the adaptation of trainees to changes imposed by the pandemic.

Another limitation is connected with the choice of using a survey with subjective and self-reported measures. The advantages are that it is quick and easily accessible. However, such measures may expose to the risk of responder bias and recall bias, which may limit their generalizability.

In addition, the demographic features of the entire cohort of Residents who received the questionnaire were not available due to privacy issues. Therefore, we could not address whether the higher rate of female respondents reflected a higher proportion of female Residents attending the Training Programs enrolled in our study or not. Although we cannot exclude the presence of biases, this percentage may also reflect the predominance of women working as physicians in Italy, that in 2019 made up 68% of the total (Italian Ministry of Health, 2019).

Finally, despite our efforts for stimulating participation, we had a low response rate of 30.3%. This might be due to different reasons, including the lack of compensation, the length of the questionnaire, and the fact that medical trainees are usually busy and work long hours. Indeed, other published studies of this kind have a similar or lower response rate, between 15% and 25% [9,14,24,52]. Because of that, caution must be used when interpreting the results.

### Strengths and perspectives

One of the main strengths of our study is represented by the inclusion of residents belonging to various specialties. Compared to previous works, which have focused their interest on specific training programs, our survey offered the opportunity to explore COVID-19 impact on a broader spectrum of Residency Programs and, consequently, to identify possible positive effects of the pandemic on trainees of certain specialties. Moreover, we investigated several aspects of residents’ competencies, satisfaction, activity, training, as well as control and infection prevention measures and quality of life, thus allowing us to depict a bigger picture of the impact of COVID-19 first wave on residency training in Italy.

These insights will be helpful not only in the current pandemic period, but also for future trainees. First of all, given the unique climatic and geographical changes that we are now witnessing, Training Programs worldwide should develop a “crisis plan” to deal with future health emergencies. Before COVID-19 pandemic, no study was available in the literature to inform Program Directors about the best way to deal with such situations. Nowadays, Directors and trainees might take advantage of the available studies to devise efficient and quick solutions. In addition, some of the strategies used during COVID-19 pandemic, such as virtual learning and telemedicine, might be here to stay. Our study points out that, while most Training Programs incorporated virtual learning in their curriculum, only half of the residents were satisfied with it. Therefore, more effort is needed to improve the quality of virtual education for residents in Lombardy.

We also found a decrease in quality of life and concerns related to COVID-19 exposure. Future studies should focus on long-term effects of pandemic-related stress and on effective countermeasures [50]. Our results could also encourage Italian Universities to improve their offer of psychological support services for medical residents.

Another crucial issue is the availability of PPE and proper surveillance for at-risk personnel. Our recommendation is to always consider medical residents as part of the hospital staff and to offer appropriate PPE and tests. Universities should make sure that hosting hospitals respect these conditions, and they should implement systems to report misconduct.

## Conclusions

Overall, our findings underscore that COVID-19 pandemic may have exerted a disproportionate impact on certain groups of HCWs such as residents in the Lombardy area. Among them, the impact of COVID-19 first wave was not homogeneous, as there was a significant disruption in procedure-based training, while a positive impact is reported by the majority of residents involved in COVID and urgent care. Educational activities were also deeply affected by the pandemic, and while most training programs adopted virtual teaching methods, residents were not completely satisfied with these strategies. These findings should prompt a response from Universities and Residency Programs, and an effort should be made to make up for the training lost in those months.

Another important issue to be considered is the dramatic impact of COVID-19 on residents’ quality of life, which appears significantly worsened in five key areas. Moreover, a vast proportion of residents reported a high occupational exposure to SARS-CoV-2, and a considerable proportion considered themselves at higher risk of COVID-19 infection compared to senior staff. As we transition into post-pandemic society, further studies are needed to bridge the gap between providing optimal medical education and training and preserving the well-being of future physicians.

## Supplementary Material

Supplemental Material

## Data Availability

The data that support the findings of this study are available on request from the corresponding author (Elena Abati, e-mail: elena.abati@unimi.it). The data are not publicly available due to privacy reasons.
