# Supplementary material for: A cross-sectional survey study of the impact of COVID-19 pandemic on the training and quality of life of Italian medical residents in the Lombardy region
Source: Ann Med. 2022 Aug 24;54(1):2326–39. doi: 10.1080/07853890.2022.2105392 (PMC9415486; doi:10.1080/07853890.2022.2105392)
Supplement: Supplemental Material [file IANN_A_2105392_SM7798.pdf]

Supplementary Materials

Appendix 1 – Survey "Impact of SARS-CoV-2 on the training and quality of life of Italian Medicine and Surgery Residents" (English translation)

Section 1: Demographics

1. Are you currently enrolled in a Postgraduate School of Medicine and Surgery? Yes/no

2. If you answered yes to the previous question, which Postgraduate School are you enrolled in? (list of schools)

3. In which University do you attend the Specialization course? University of Brescia, Brescia / University of Milan, Milan / University "Vita-Salute San Raffaele", Milan / University of Pavia, Pavia / Other (please specify)

4. Which course year are you enrolled in? First, Second, Third, Fourth, Fifth

5. How old are you? (answered in whole numbers)

6. Gender?

7. What city do you work in?

8. In which hospital did you work before February 20, 2020?

9. In which hospital have you worked from February 20, 2020 to now? The same as before; another hospital (please specify which hospital).

10. Citizenship: choose from list

11. Marital status: single / single, married, separated / divorced, widower

12. How many children do you have?

13. Monthly family income: <1500, 1500-2500, 2500-3500, 3500-4500,> 4500

14. If you are a woman, are you pregnant? Yes/No

15. Comorbidity Yes / No.

16. If Yes, please specify: .....

17. Do you smoke tobacco or cigarettes, even occasionally? Yes/no

For Peer Review Only

18. If you answered yes to the previous question, how often have you smoked tobacco or cigarettes in the past 30 days? Every day/Several times a week/Once a week/2-3 times a month/Only once

19. Do you smoke electronic cigarettes, even occasionally? Yes/no

20. If you answered yes to the previous question, how often have you smoked e-cigarettes in the past 30 days? Every day/Several times a week/Once a week/2-3 times a month/Only once

21. Do you consume alcohol? No, never/Yes, once a month or less/Yes, about 2-4 times a month/Yes, about 2-3 times a week/Yes, 4 or more times a week

22. Are you currently outside the training network (abroad or in facilities / hospitals in Italy outside the training network)? Yes/no

Section 2: Clinical Activity

23. Do you carry out clinical activities within your Postgraduate School? Yes/No →refer to next section

24. What type of clinical activity did you deal with from the start of your specialist training to February 20, 2020 (you can select more than one answer)?

● Inpatient clinic

● Outpatient clinic in-hospital

● Day Hospital

● Outpatient clinic outside the hospital

● Emergency Department

● Other:

25. How satisfied were you with the clinical activity you performed in the Postgraduate School before February 20, 2020? Express a rating on a satisfaction scale from 1 to 5 (1 = very dissatisfied; 2 = dissatisfied; 3 = neutral (neither satisfied nor dissatisfied); 4 = satisfied; 5 = very satisfied)

26. What type of clinical activity have you been involved in from February 20, 2020 to today? You can select more than one answer.

● Specialist Inpatient clinic

● COVID inpatient clinic

● Outpatient clinic in-hospital

● Day Hospital

● Outpatient clinic outside the hospital

● Emergency Department

● Other:

27. What was the impact of the SARS-CoV-2 pandemic on the clinical activity of the ward where you worked in general? Highly increased / slightly increased / unchanged / slightly decreased / greatly decreased

28. What has been the impact of the SARS-CoV-2 pandemic on your clinical activity in the ward in quantitative terms? Highly increased / slightly increased / unchanged / slightly decreased / greatly decreased

29. If it declined, for which reason (you can select more than one answer)?

● Reduced number of hospitalizations / reduction of beds

● Total or partial conversion to the COVID department

● Reduction in the number of doctors admitted to the ward for reasons of contagion containment

● Scarcity / absence of PPE

● Other:

30. If it increased, for what reason (you can select more than one answer)?

● Increase in the number of hospitalizations or beds

● total or partial conversion to the covid department

● reduction of staff available due to contagion among staff

● increase in the frequency or length of shifts

● Other:

31. What has been the impact of the SARS-CoV-2 pandemic on your outpatient clinical activity in quantitative terms? Highly increased / slightly increased / unchanged / slightly decreased / greatly decreased/I was not involved in outpatient clinical activity

32. What has been the impact of the SARS-CoV-2 pandemic on your clinical day hospital operation in quantitative terms? Highly increased / slightly increased / unchanged / slightly decreased / greatly decreased/I was not involved in Day Hospital activities

33. Have your clinical duties changed since February 20, 2020? if so, how:

● No, I continued to carry out the same type of clinical activity

For Peer Review Only

- Yes, I have been relocated to a COVID department
- Yes, I was relocated to a non-COVID ward other than mine
- Yes, I have been relocated to PS / triage activities
- Yes, I was asked to carry out activities that did not involve contact with patients (switchboard, bureaucracy, etc.)
- Yes, I was hired through the regional call in a COVID department other than mine
- Yes, I was hired through the regional call in my department
- Other:

34. If your duties have changed, have you volunteered for it or have you been forced by your chief / tutor?

- I volunteered
- It was decided by my chief / tutor

35. How satisfied are you with your clinical activity from March 2020 onwards? Express a rating on a satisfaction scale from 1 to 5 (1 = very dissatisfied; 2 = dissatisfied; 3 = neutral (neither satisfied nor dissatisfied); 4 = satisfied; 5 = very satisfied)

36. What do you think the impact of the COVID-19 pandemic has been on your clinical abilities on a scale of 1 to 5 (1 = greatly worse; 2 = slightly worse; 3 = unchanged; 4 = slightly improved; 5 = greatly improved)?

Section 3: Surgical activity

37. Do you carry out surgical activities within your Postgraduate School? Yes, No -> refer to the next section

38. How satisfied were you with the surgical activity you performed in the Specialization School before February 20, 2020? Express a rating on a satisfaction scale from 1 to 5 (1 = very dissatisfied; 2 = dissatisfied; 3 = neutral (neither satisfied nor dissatisfied); 4 = satisfied; 5 = very satisfied)

39. What was the impact of the SARS-CoV-2 pandemic on the surgical activity of the ward in which you worked in general? Highly increased / slightly increased / unchanged / slightly decreased / greatly decreased

40. What has been the impact of the SARS-CoV-2 pandemic on your surgical activity in quantitative terms? Highly increased / slightly increased / unchanged / slightly decreased / greatly decreased

For Peer Review Only

41. If it declined, why (you can select more than one answer)?

● Closure of the ward or reduction of beds

● I have been relocated to a non-surgical ward

● Reduction of staff in the room for reasons of containment of the contagion

● Reduction of staff in the room due to lack / absence of PPE

● I was hired by the Lombardy Region in the covid department

● Other:

42. If it increased, for what reason (you can select more than one answer)?

● I work in a hub center for my discipline

● Reduction of staff due to contagion among staff

● Increase in length or frequency of shifts

● Increase in the number of interventions

● Reduction of staff due to relocation of part of it in non-surgical wards

● Other:

43. How satisfied are you with your surgical activity from February 20, 2020 onwards? Express a rating on a satisfaction scale from 1 to 5 (1 = very dissatisfied; 2 = dissatisfied; 3 = neutral (neither satisfied nor dissatisfied); 4 = satisfied; 5 = very satisfied)

47. What do you think the impact of the COVID-19 pandemic has been on your surgical skills on a scale of 1 to 5 (1 = greatly worse; 2 = slightly worse; 3 = unchanged; 4 = slightly improved; 5 = greatly improved)?

Section 4: Research activities

44. Do you carry out research activities within your Residency Program? Yes, No → refer to next section

45. What kind of research activities do you carry out or did you carry out?

● Clinical research

● Basic research

For Peer Review Only

46. How satisfied were you with the research you carried out within the Postgraduate School before February 20, 2020? Express a rating on a satisfaction scale from 1 to 5 (1 = very dissatisfied; 2 = dissatisfied; 3 = neutral (neither satisfied nor dissatisfied); 4 = satisfied; 5 = very satisfied)

47. What was the impact of the SARS-CoV-2 pandemic on the research activities of the laboratory / team you worked in? Highly increased / slightly increased / unchanged / slightly decreased / greatly decreased

48. What has been the impact of the SARS-CoV-2 pandemic on your research activity in quantitative terms? Highly increased / slightly increased / unchanged / slightly decreased / greatly decreased

49. How satisfied are you with your research activity from 20 February 2020 until now? Express a rating on a satisfaction scale from 1 to 5 (1 = very dissatisfied; 2 = dissatisfied; 3 = neutral (neither satisfied nor dissatisfied); 4 = satisfied; 5 = very satisfied)

50. What do you think the impact of the COVID-19 pandemic has been on your research activities on a scale of 1 to 5 (1 = significantly worse; 2 = slightly worse; 3 = unchanged; 4 = slightly improved ; 5 = greatly improved)?

51. Have you carried out research activities focused on COVID19? Yes/No.

Section 5: Activities abroad or outside the training network

52. Were you carrying out a period abroad or outside the training network at the time of the appearance of the first cases of SARS-CoV-2 in Italy (February 2020)? Yes, No → refer to next section

53. Specifically, where were you?

● European Union

● Foreign country not belonging to the EU

● Hospital / research center in Italy

54. What was the impact of the SARS-CoV-2 pandemic on the activities of the hosting facility where you worked? Highly increased / slightly increased / unchanged / slightly decreased / greatly decreased

55. What has been the impact of the SARS-CoV-2 pandemic on your hosting facility in quantitative terms? Highly increased / slightly increased / unchanged / slightly decreased / greatly decreased

56. Did you return to the training network after the onset of the pandemic? Yes/no

57. If you answered yes to the previous question, why? (you can select more than one option)

For Peer Review Only

- I was asked to return by my Program Director / Tutor
- I chose to return to help colleagues during the pandemic
- I chose to return for personal or family reasons
- I chose to return for fear of contagion
- I chose to return because the facility where I was ceased or reduced its activity
- Other:

Section 6: Education and training

58. Did you attend classes (lectures, seminars, etc.) before February 2020 within your Postgraduate School? Yes, no, I don't know

59. Were there any practical training activities (e.g. professional traineeships in specialist Units) planned before February 2020 within your Specialization school? Yes, no, I don't know

60. Were classes (lectures, seminars, etc.) carried out after February 2020 within your Specialization School? Yes/No

61. Were practical training activities (eg professional traineeships in specialist areas) carried out after February 20, 2020 within your Specialization School? Yes/No

62. What was the impact of the SARS-CoV-2 pandemic on your classes in quantitative terms? Highly increased / slightly increased / unchanged / slightly decreased / greatly decreased

63. What was the impact of the SARS-CoV-2 pandemic on your practical training activities in quantitative terms? Highly increased / slightly increased / unchanged / slightly decreased / greatly decreased

64. How satisfied were you with the teaching and training activities provided by your Specialization School before February 20, 2020? Express a rating on a satisfaction scale from 1 to 5 (1 = very dissatisfied; 2 = dissatisfied; 3 = neutral (neither satisfied nor dissatisfied); 4 = satisfied; 5 = very satisfied)

65. How satisfied are you with the teaching and training activities provided by your Postgraduate School from March 2020 onwards? Express a rating on a satisfaction scale from 1 to 5 (1 = very dissatisfied; 2 = dissatisfied; 3 = neutral (neither satisfied nor dissatisfied); 4 = satisfied; 5 = very satisfied)

66. Has your Postgraduate School adopted distance learning methods from February 20, 2020? Yes/No

For Peer Review Only

67. If you answered yes to the previous question, which type of distance learning was adopted? (You can select more than one answer)

- Webinars
- Lectures through virtual platforms (eg. Microsoft Teams, Google Meets, etc.)
- Powerpoint presentations pre-recorded by teachers and uploaded to sharing platforms
- External teaching materials (eg websites, online courses ..)
- Participation in virtual congresses
- Presentation of insights by trainees
- Other:

68. If you answered yes to question no.66, how satisfied are you with the distance learning methods adopted by your Residency School from February 20, 2020 onwards? Express a rating on a satisfaction scale from 1 to 5 (1 = very dissatisfied; 2 = dissatisfied; 3 = neutral (neither satisfied nor dissatisfied); 4 = satisfied; 5 = very satisfied)

69. What do you think the impact of the SARS-CoV-2 pandemic has been on your educational training on a scale of 1 to 5 (1 = greatly worsened; 2 = slightly worsened; 3 = unchanged; 4 = improved by little; 5 = much improved)?

Section 7: Health surveillance and contagion prevention

70. In the department where you work, are COVID-19 nasopharyngeal swabs available for trainees? Yes, no, I don't know

71. If yes, who can access them? (You can select more than one answer)

- trainees who have had direct contact with confirmed cases of covid-19
- Trainees working with covid-19 + patients
- Trainees with symptoms compatible with Covid-19
- All trainees
- Other ....

72. Has your facility provided for the performance of serology screening for trainees? Yes, no, I don't know

For Peer Review Only

73. If you answered yes to the previous question, have you already taken the serological test? Yes, No, I will do it in the next few days.

74. Did you have access to PPE (sterile gloves, water-repellent gowns, surgical masks / FFP2-3 masks, visors, overshoes) adequate to the level of risk of the activities you performed?

● Yes, always

● Not always but on most occasions (> 75% of the time)

● Occasionally (between 25% and 75% of the time)

● Sporadically (<25%)

● Never

75. If you work in a COVID-19 ward, have you undergone training on patient management or the use of ventilation devices?

● Yes, directly in the ward

● Yes, remotely

● No, I studied on my own

● No

76. Have you been in direct contact with SARS-CoV-2 positive patients or colleagues? Yes, no, I don't know

77. If so, have you communicated it to the organization you work for? Yes/No

78. If you answered yes to the previous question, what was the protocol put into practice by the structure you work for? (you can select more than one answer)

● I was advised to self-isolate as a precaution regardless of the appearance of symptoms

● I took a nasopharyngeal swab

● I performed a serological study

● I was advised to self-isolate only in case of symptoms

● No preventative measure was adopted

79. Have you experienced symptoms attributable to COVID-19 since the start of the pandemic?

● No

● Yes, mild

For Peer Review Only

● Yes, moderate / severe but managed at home

● Yes, I was hospitalized

80. If so, have you tested positive for COVID-19?

● Yes

● No

● No swab was performed

81. Have you ever had the feeling that your trainee status has put you at greater risk of contracting COVID during your work? Yes/No

82. Have you ever had the feeling that PPE was not administered fairly to the trainees compared to other physicians working for your hospital?

● This has never happened

● This has hardly ever happened

● It has happened a few times

● It happened quite often

● It always happened

Section 9: Quality of life

83. Do you live with other people?

● Yes, with my family

● Yes, with my / my partner

● Yes, with roommates

● No

84. Have you taken the following precautionary measures to avoid putting your cohabitants at risk? (You can select more than one answer)

● Change your home address

● Sleeping in separate rooms (if not already in place before the pandemic)

● Using personal disinfection methods when returning from work (eg shower)

For Peer Review Only

● Using disinfection methods for personal items upon returning from work (sanitizing personal items, changing and washing clothes, etc.)

● Other: …

Before February 20, 2020, how would you rate the following:

85. How would you rate the quality of your sleep? (1 = very dissatisfied; 2 = dissatisfied; 3 = neutral (neither satisfied nor dissatisfied); 4 = satisfied; 5 = very satisfied)

86. How would you rate the quality of your mood? (1 = very dissatisfied; 2 = dissatisfied; 3 = neutral (neither satisfied nor dissatisfied); 4 = satisfied; 5 = very satisfied)

87. How would you rate the quality of your family relationships? (1 = very dissatisfied; 2 = dissatisfied; 3 = neutral (neither satisfied nor dissatisfied); 4 = satisfied; 5 = very satisfied)

88. How would you rate the quality of your social relationships? (1 = very dissatisfied; 2 = dissatisfied; 3 = neutral (neither satisfied nor dissatisfied); 4 = satisfied; 5 = very satisfied)

89. How would you rate your job satisfaction? (1 = very dissatisfied; 2 = dissatisfied; 3 = neutral (neither satisfied nor dissatisfied); 4 = satisfied; 5 = very satisfied)

From February 20 to now, how would you rate the following:

90. How would you rate the quality of your sleep? (1 = very dissatisfied; 2 = dissatisfied; 3 = neutral (neither satisfied nor dissatisfied); 4 = satisfied; 5 = very satisfied)

91. How would you rate the quality of your mood? (1 = very dissatisfied; 2 = dissatisfied; 3 = neutral (neither satisfied nor dissatisfied); 4 = satisfied; 5 = very satisfied)

92. How would you rate the quality of your family relationships? (1 = very dissatisfied; 2 = dissatisfied; 3 = neutral (neither satisfied nor dissatisfied); 4 = satisfied; 5 = very satisfied)

93. How would you rate the quality of your social relationships? (1 = very dissatisfied; 2 = dissatisfied; 3 = neutral (neither satisfied nor dissatisfied); 4 = satisfied; 5 = very satisfied)

94. How would you rate your job satisfaction? (1 = very dissatisfied; 2 = dissatisfied; 3 = neutral (neither satisfied nor dissatisfied); 4 = satisfied; 5 = very satisfied)

95. In this section it is possible to leave comments to the researchers of this study:

For Peer Review Only

Supplementary Tables

Table S1 – Additional sociodemographic characteristics of surveyed Italian residents.

Characteristic	No. (%) (n=498)
**Pregnancy**	4 (1.3% of female population)
**Children (≥1)**	23 (4.6%)
**Civil status**	
**Single or divorced**	441 (88.6%)
**Married**	57 (11.4%)
**Monthly family income§**	
**<2500 €**	320 (64.3%)
**2500-3500 €**	103 (20.7%)
**>3500 €**	75 (15.1%)
**Presence of Comorbidities†**	46 (9.2%)
**Cigarette smoking**	114 (22.9%)
**E-Cigarette smoking**	18 (3.6%)
**Frequency of alcohol consumption**	
**Never or 1 in a month**	100 (20.1%)
**2-4 in a month**	236 (47.4%)
**>=2 in a week**	162 (32.5%)
**Residents involved in clinical activity in their training program (n= 471)**	430 (91.3%)
**Involvement in specific activities before COVID-19 pandemic**	
**Inpatient care**	372 (86.5%)
**Outpatient care (in-hospital)**	262 (60.9%)
**Outpatient care (out of the hospital)**	20 (4.7%)
**Day Hospital**	130 (30.2%)
**Emergency Room**	157 (36.5%)
**Involvement in specific activities during COVID-19 pandemic**	
**Inpatient care**	272 (63.3%)
**Outpatient care (in-hospital)**	141 (32.8%)
**Outpatient care (out of the hospital)**	15 (3.5%)
**Day Hospital**	54 (12.6%)
**Emergency Room**	114 (26.5%)
**COVID-19 pre-triage activity**	140 (32.6%)
**Residents involved in surgical activity in their training program (Total n= 471)**	122 (25.9%)

For Peer Review Only

**Residents involved in research activity in their training program (n= 471)**	288 (61.1%)
**Basic research**	69 (24%)
**Clinical research**	254 (88.2%)
**Residents attending a different institution for a training period outside from home University at the onset of COVID-19 pandemic (n= 498)**	24 (4.8%)
**Residents attending an Italian Institution**	5 (20.8%)
**Residents attending a European Institution**	14 (58.3%)
**Residents attending a non-European Institution**	5 (20.8%)
**Residents attending a different institution for a training period outside from home University at the time of survey completion (n= 498)**	27 (5.1%)
**Training received by residents before COVID-19 pandemic (n= 498)**	
**Frontal lessons**	458 (92%)
**Practical training**	306 (61.4%)

†*Main comorbidities include asthma (27.9%), hypothyroidism (13.9%) and cardiac valvulopathy (7.0%)*

§Values were chosen based on mean monthly family incomes in Northern Italy, which, according to the 2019 Annual report of the Italian National Institute of Statistics (ISTAT, Istituto Nazionale di Statistica), amount to roughly 2.900 euros per family. Based on these values, we decided to stratify responses according to the three brackets shown.

For Peer Review Only

Table S2. Impact of COVID-19 pandemic on residents' clinical, surgical and research activities and on training programs among surveyed residents in Italy during the first wave.

	Increased	Decreased	Unchanged
**Clinical activity in inpatient clinic** (n=398)	193 (48.5%)	166 (41.7%)	39 (9.8%)
**Clinical activity in outpatient clinic** (n=205)	24 (9.3%)	210 (81.1%)	25 (9.7%)
**Clinical activity in Day Hospital service** (n=166)	13 (7.8%)	128 (77.1%)	25 (15.1%)
**Surgical activity** (n=122)	5 (4.1%)	108 (88.5%)	9 (7.4%)
**Research activity** (n=288)	160 (55.6%)	85 (29.5%)	43 (14.9%)
**Activity at hosting Institution abroad** (n=24)	5 (20.8%)	15 (62.5%)	4 (16.7%)
**Frontal lessons provided by Training Programs** (n=498)	16 (3.2%)	444 (89.2%)	38 (7.6%)
**Practical training provided by Training Programs** (n=498)	28 (5.6%)	355 (71.3%)	115 (23.1%)

<u>Variations in clinical activity</u>:

Reasons for decrease (n=166) (>1 answer allowed)

- **Reduction in hospitalizations/beds** (n=127, 76.5%)
- **Conversion to COVID-ward** (n=60, 36.1%)
- **Limitation of trainees' number per shift to reduce exposure** (n=44, 26.5%)

Reasons for increase (n=193) (>1 answer allowed)

- **Conversion to COVID-ward** (n=145, 75.1%)
- **Increase in shifts' duration and number** (n=104, 53.9%)
- **Increase in hospitalizations/beds** (n=103, 53.4%)
- **Reduction of personnel due to contagion** (n=66, 34.2%)
- **Conversion to Hub centre** (n=50, 25.9%)

Change (n=430)

- **Yes** (n=272, 63.3%)
- **No** (n=158, 36.7%)

Reasons for change (n=272)

- **Deployment to COVID-ward** (n=152, 55.9%)
- **Deployment to administrative services** (n=70, 25.7%)
- **Deployment to different non-COVID-ward** (n=22, 8.1%)
- **Hiring in COVID and non-COVID-ward** (n=14, 5,1%)
- **Deployment to ER/triage activity** (n=10, 3.7%)

Was the change voluntary or imposed? (n=272)

- **Imposed** (n=140, 51.4%)
- **Voluntary** (n=132, 48.6%)

For Peer Review Only

<u>**Variations in surgical activity**</u>:

Reasons for decrease (n=108)

- **Ward closure or reduction in hospitalizations/beds** (n=94, 87%)
- **Limitation of trainees' number in OR** (n=41, 38%)
- **Deployment to COVID ward** (n=24, 23%)

Reasons for increase (n=5)

- **Reduction of personnel due to contagion** (n=3, 60%)
- **Increase in surgical interventions** (n=1, 20%)
- **Increase in shifts' duration and number** (n=1, 20%)

<u>**Variations in research activity**</u>:

Reasons for decrease (n=85)

- **Reduction in the number of ongoing research projects** (n=59, 69.4%)
- **Reduction in dedicated research time** (n=36, 42.4%)
- **Increased surgical or clinical duties** (n=22, 25.9%)
- **Quarantine** (n=12, 14.1%)

Reasons for increase (n=160)

- **Establishment of novel COVID-related research projects** (n=132, 82.5%)
- **Increase in the number of ongoing research projects** (n=77, 48.1%)
- **Increase in dedicated research time** (n=71, 44.4%)

<u>**Variations in the activity at hosting Institution abroad**</u>:

Residents returning to home University during COVID-19 pandemic (n=13, 54.2%)

Reasons for returning to home University (n=13)

- **Personal or family reasons** (n=9, 69.2%)
- **To help colleagues during pandemic** (n=5, 38.5%)
- **Request of Program Director** (n=1, 7.7%)
- **Safety concerns** (n=1, 7.7%)

<u>**Variations in the Training Program**</u>:

Residents receiving remote training during COVID-19 pandemic (n=391, 78.5%)

Type of remote training provided (n=391) (>1 answer allowed)

- **Frontal lessons on virtual platforms** (n=349, 89.3%)

For Peer Review Only

Webinars (n=140, 35.8%)

Pre-registered Powerpoint presentations (n=127, 32.5%)

Virtual presentations prepared by Residents (n=70, 17.9%)

Participation to virtual congresses (n=62, 15.9%)

Online courses and/or websites (n=52, 13.3%)

Abbreviations: COVID-19, Coronavirus disease-2019; ER, emergency room; OR, operation room; PPE, personal protective equipment.

For Peer Review Only

Supplementary Table S3 – Health surveillance and infection prevention measures adopted by health-care institutions during first COVID-19 pandemic wave in Italy as reported by surveyed residents.

**Characteristic**	**No. (%)** (n=498)
**Availability of SARS-CoV-2 Nasopharyngeal Swab Testing**	150 (30.1%)
**SARS-CoV-2 Nasopharyngeal Swab Testing Criteria** (n=150)	
Direct contact with Covid-19 cases	64 (42.7%)
Attending Covid-19 patients	49 (32.6%)
Symptomatic for Covid-19 disease	61 (40.6%)
All above criteria	65 (43.3%)
Other	2 (1.3%)
**Availability of SARS-CoV-2 serology assay**	413 (82.9%)
**SARS-CoV-2 serology assay performed** (n=413)	396 (95.9 %)
**PPE availability**	
Never/<25% of time	32 (6.4 %)
25-75% of time	53 (10.6 %)
>75% of time/Always	413 (82.9 %)
**If working with Covid-19 patients, training for management of Covid-19 and mechanical ventilation (remotely or in-hospital)** (n=324)	129 (39.8%)
**Contact with Covid-19 cases**	355 (71.3 %)
**Isolation precautions implemented after being in contact with a suspected/confirmed COVID-19 case** (n=355)	
Quarantine isolation period	40 (11.2 %)
Quarantine isolation period only if symptomatic	113 (31.8 %)
SARS-CoV-2 nasopharyngeal swab	132 (37.1 %)
SARS-CoV-2 serology assay	24 (6.7 %)
No intervention	109 (30.7 %)
**Covid-19 symptoms**	
Yes, mild	83 (16.7 %)
Yes, moderate/severe	16 (3.2 %)
**If Covid-19 symptoms, SARS-CoV-2 nasopharyngeal swab performed** (n=99)	77 (75.6 %)
**If Covid-19 symptoms and SARS-CoV-2 nasopharyngeal swab, test positivity** (n=77)	20 (26.0 %)
**Household composition**	
Living alone	102 (20.5%)
Living with partner	217 (43.5%)

For Peer Review Only

Living with family	97 (19.5%)
Living with flatmate/s	82 (16.5%)
**Strategies for contagion prevention at home, if not living alone (more than one answer is allowed)** (n=396)	
Moving elsewhere	50 (12.6%)
Sleeping in separate bedroom	8 (2%).
Personal disinfection/hygiene	278 (70%)
Personal items disinfection	268 (68%)

Abbreviations: HCW, healthcare workers; PPE, personal protective equipment.

For Peer Review Only
